# Analysis of the Morphological Characteristics of the Palatal Rugae for Three-Dimensional Superimposition of Digital Models in Korean Subjects

**DOI:** 10.1155/2018/3936918

**Published:** 2018-11-26

**Authors:** Sung-Hwan Choi, Kyongmin Koh, Kee-Joon Lee, Chung-Ju Hwang, Jung-Yul Cha

**Affiliations:** ^1^Assistant Professor, Department of Orthodontics, Institute of Craniofacial Deformity, Yonsei University College of Dentistry, Seoul, Republic of Korea; ^2^Graduate Student, Department of Orthodontics, Yonsei University College of Dentistry, Seoul, Republic of Korea; ^3^Professor, Department of Orthodontics, Institute of Craniofacial Deformity, Yonsei University College of Dentistry, Seoul, Republic of Korea; ^4^Professor, Department of Orthodontics, Institute of Craniofacial Deformity, Yonsei University College of Dentistry, Yonsei University, Seoul, Republic of Korea

## Abstract

**Objective:**

The aim of this study was to evaluate the morphological characteristics of the palatal rugae in Korean subjects to determine whether the palatal rugae can be used as an appropriate reference area for three-dimensional digital model superimpositions.

**Materials and Methods:**

In total, 343 patients (110 men, 233 women; mean age, 25.6±8.2 years) who had a digital model taken at their initial visit were included, and the numbers and types of right and left palatal rugae were investigated according to the primary, secondary, and fragmentary rugae. Finally, the differences in the positions of the third primary ruga were investigated according to the presence of additional rugae posterior to the third primary ruga.

**Results:**

The number of primary palatal rugae ranged from one to six, with 43.5% of the subjects having three primary rugae and 36.1% having four primary rugae; there were no significant differences between sexes. Except for the fragment rugae, the numbers of primary and secondary rugae were not significantly different between the left and right sides. The third primary ruga was located more significantly anteriorly when there was an additional ruga posterior to the third primary ruga (*P* < 0.001).

**Conclusions:**

The numbers of the palatal rugae vary greatly among individuals, and this affects the anteroposterior position of the third primary ruga. When the third primary ruga is located anteriorly, care should be taken when using it as a reference area for superimposition with a digital model before and after orthodontic treatment.

## 1. Introduction

In current dental practice, digital models are rapidly replacing conventional dental casts to improve storage and data accessibility. A three-dimensional (3D) digital model can be obtained by scanning the actual oral cavity or dental casts with laser beams. Previous studies have compared the accuracy of digital models and conventional dental casts in measuring linear distances [1], including the width [2], height [3], overjet and overbite [4], and intermolar and intercanine width of teeth [3], and they have found that digital model measurements are almost identical to those of conventional dental casts [5–7].

Recently, a method that uses 3D models to analyze the pre- and posttreatment movements of teeth after orthodontic or orthognathic surgery has been introduced to quantitatively measure tooth movements during treatment [8–10]. By superimposing the pre- and posttreatment digital models, the vertical, horizontal, and anteroposterior movements of individual teeth, as well as their rotation, can be determined. In addition, this method can be used to analyze changes in arch width after treatment [11, 12].

The stable dental structures of digital models during orthodontic treatment have been examined to identify a stable reference region for the superimposition of maxillary digital models. The palatal rugae are irregular ridges situated in the anterior part of the palatal mucosa behind the incisive papilla [13]. Since the palatal rugae patterns have different characteristics for each individual and typically change very little over a lifetime, the palatal rugae patterns have been commonly used as a reference area for superimposition before and after orthodontic treatment; it has been also used in forensic identification for individuals, similar to the use of fingerprints [14, 15].

However, although controversial, several previous studies have reported that the medial and lateral ends of the palatal rugae adjacent to anterior teeth tend to follow the movement of the teeth in the anteroposterior and mediolateral directions, especially after orthodontic treatment when the amount of retraction of the maxillary anterior teeth is large through the premolar extraction space [8, 10, 16, 17]. Recent studies have suggested that if the 3rd primary ruga is located far from the anterior teeth, i.e., near the first molar, it is possible to use the medial part of the 3rd primary ruga and part of the palatal vault as the reference area [18–20]. Nevertheless, no consensus exists on the area upon which digital models should be superimposed for orthodontic treatment.

To date, despite reports showing that morphological characteristics of the palatal rugae vary ethnically and regionally, there has been a lack of 3D studies on the anatomical features of the palatal rugae (number, distribution, and position of the palatal rugae, especially the 3rd primary ruga) in Korean subjects [20–22]. If the 3rd primary ruga is located anteriorly at a higher rate than expected, it is more likely to undergo a change in the anterior-posterior position due to orthodontic tooth movement, and it would be inappropriate for use as a reference area.

Therefore, the aim of this study was to evaluate the morphological characteristics of the palatal rugae in Korean subjects to determine whether the palatal rugae can be used as an appropriate reference area for 3D digital model superimpositions.

## 2. Materials and Methods

### 2.1. Samples

To analyze the anatomical features of the palatal rugae, 343 patients (110 men, 233 women; mean age, 25.6±8.2 years) who visited the Department of Orthodontics, Yonsei University College of Dentistry, between January 2009 and May 2016 were selected according to the following inclusion criteria: a digital model taken at the initial visit was available, patient age was at least 17 years, patient had permanent dentition, with no maxillofacial deformities, no congenital defects of the teeth from the maxillary incisors to the second molars, no impacted teeth, no history of orthodontic treatment and/or orthognathic surgery, and mild crowding less than 4 mm. This study followed the guidelines of the Declaration of Helsinki and was approved by the institutional review board of Yonsei University Dental Hospital.

### 2.2. Model Scanning

Impressions for maxillary dental casts were taken using alginate impression material at the initial visit. Dental casts were made with dental stone and then scanned by an Orapix 3D scanner (laser slit-type noncontact 3D scanner, Orapix Co. Ltd., Seoul, Republic of Korea; accuracy ±20 *μ*m), and the data were stored as stereolithography files. The following parameters were measured using a 3D reverse modeling software program (Rapidform 2006, INUS Technology Inc., Seoul, Republic of Korea).

### 2.3. Measurements

#### 2.3.1. Classification and Distribution of the Palatal Rugae

**Figure 1:**
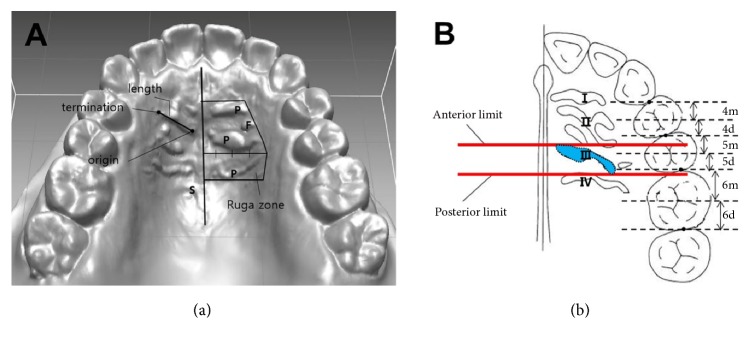
Analysis of the anatomical features of the palatal rugae. (a) Classification of the ruga. P: primary ruga; S: secondary ruga; F: fragmentary ruga. The box zone: ruga zone. (b) Measurement of the anterior limit and posterior limit of the 3rd ruga.

We classified the palatal rugae into the following three types: primary, secondary, and fragmentary (Figure 1(a)) [20]. The following definitions were used:

(i) Definition of the ruga zone: the medial boundary of the rugae was defined as the midline of the hard palate. The lateral boundary was the point of termination of the most lateral ruga. The anterior boundary was defined as the anterior aspect of the primary ruga, and the posterior boundary was the posterior aspect of the ruga closest to the 1st molar.

(ii) Primary ruga: the primary ruga was at least 5 mm in length and highly prominent, and it originated in the medial three-quarters of the ruga zone.

(iii) Secondary ruga: the secondary ruga was at least 3 mm and less than 5 mm in length and less prominent than the primary ruga.

(iv) Fragmentary ruga: the fragmentary ruga was at least 2 mm and less than 3 mm in length.

The numbers of primary, secondary, and fragmentary palatal rugae on the right and left sides were measured for each subject according to the above-mentioned classification system.

#### 2.3.2. Position of the 3rd Primary Ruga

The anterior and posterior limits of the 3rd primary ruga were measured in the digital model (Figure 1(b)). Lines that were perpendicular to the median line and that passed through the interdental contact points, as well as lines that bisected each tooth, were drawn. The part of each tooth that was anterior to the bisecting line was defined as m, and the posterior part of the tooth was defined as d. The anterior limit of the third primary ruga was delimited by the line that was perpendicular to the median line, and the most anterior point of the 3rd primary ruga was recorded in relation to the teeth. The posterior limit of the 3rd primary ruga was delimited in the same manner. In addition, based on the contact points between the first premolar and the second premolar, we assessed whether there was a difference in the position of the anterior or posterior limit of the 3rd primary ruga according to the presence of additional rugae posterior to the 3rd primary ruga.

All assessments were done by two observers at the same time and the scores for classification and distribution of the palatal rugae was made in consensus. To test the reliability of the method, 30 models were selected at random and the same assessment procedure was followed over a 3-week interval.

### 2.4. Statistical Analysis

All statistical analyses were performed with SPSS software for Windows (version 21.0; IBM Corp., Armonk, NY, USA). The intraclass correlation coefficient was performed for testing the reliability. The mean numbers of primary, secondary, and fragmentary palatal rugae on the left and right sides were compared with a paired *t* test. The mean numbers of primary, secondary, and fragmentary palatal rugae between sexes were compared with an independent *t* test. A chi-square test was performed to compare the frequency of the right and left primary rugae and the position of the anterior or posterior limit of 3rd primary ruga according to the presence of additional rugae posterior to the 3rd primary ruga between the right and left sides. A *P* value of 0.05 was considered significant.

## 3. Results

### 3.1. Error of the Method

The intraclass correlation coefficients were 0.97 for classification and 0.98 for distribution of the palatal rugae between the original and the repeated examinations.

### 3.2. Distribution of the Palatal Rugae

The mean number of primary rugae on the right side was 3.5±0.8 and that on the left side was 3.4±0.6 (Table 1). The numbers of primary and secondary rugae were not significantly different between the right and left sides, but the number of fragment rugae was significantly different: 1.0±0.9 on the right side and 1.3±1.2 on the left side (*P* < 0.001).

The mean number of primary and fragmentary rugae was not significantly different between sexes, but the number of secondary rugae was significantly different: 1.1±0.6 for men and 1.3±0.6 for women (*P* = 0.024) (Table 2).

The number of primary palatal rugae ranged from one to six, with 43.5% of the subjects having three primary rugae and 36.1% having four primary rugae (Table 3); 11.1% of the patients had two or fewer primary palatal rugae. There was no statistically significant difference in the frequency of primary rugae between the right and left sides.

### 3.3. Position of the 3rd Primary Palatal Ruga

Among the patients with at least three primary palatal rugae, the frequency of the occurrence of another ruga posterior to the 3rd primary ruga was investigated. The number of patients with at least one primary or secondary ruga posterior to the 3rd primary ruga was 242 (78%) on the right side and 214 (72%) on the left. The frequency of the occurrence of another ruga posterior to the 3rd primary ruga did not differ significantly between the right and left sides.

**Figure 2:**
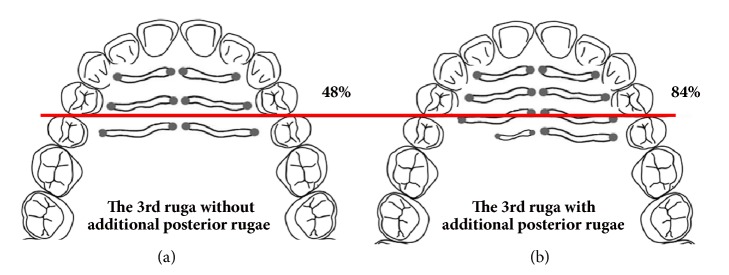
Relative position of the 3rd primary ruga in relation to the contact points of the first premolar and the second premolar. (a) Only the 3rd primary ruga; (b) the presence of additional posterior rugae. In total, 48% of the anterior limits of the 3rd palatal rugae without additional posterior rugae were located anterior to the first premolar and second premolar contact points. However, 84% of the anterior limits of the 3rd palatal rugae with additional posterior rugae were located anterior to the contact points of the first premolar and the second premolar.

The position of the 3rd primary ruga differed significantly in the presence of an additional posterior ruga (*P* < 0.001) (Table 4). With additional posterior rugae on both sides, the anterior limit of the 3rd primary ruga was more anterior compared to cases with an absence of additional posterior rugae (Figure 2). In total, 48% of the anterior limits of the 3rd palatal ruga were located anterior to the first premolar and the second premolar contact points without additional posterior rugae. However, 84% of the anterior limits of the 3rd palatal ruga with additional posterior rugae were located anterior to the first premolar and second premolar contact points.

Similarly, the posterior limit was located more significantly anteriorly in cases with additional rugae posterior to the 3rd primary palatal ruga (*P* < 0.001).

## 4. Discussion

Various reference areas have been examined in previous studies of superimposition of digital models. However, to date, no consensus has been reached on the stable maxillary structures that are unaffected by orthodontic treatment. Cha et al. [23] suggested that in extraction cases, only the palatal vault should be used as a reference area and that a best-fit matching method needs to be applied in 3D model superimpositions. In addition, Thiruvenkatachari et al. [12] identified a mushroom-shaped area of the palate as a possible reference point in pre- and posttreatment models. However, the use of the palatal vault as the only reference area is undesirable because the palatal vault area has insufficient shape characteristics, which cause rotation errors during the superimposition procedure. Recently, methods that use the medial part of the 3rd palatal ruga and the palatal vault posterior to the 3rd palatal ruga together as the reference region have been suggested [18, 19, 24]. However, although recent studies have focused on the 3rd palatal ruga as a reference point in digital superimpositions, few studies have examined the morphological features, frequency, and distribution of the palatal rugae, especially the anteroposterior position of the 3rd palatal ruga in South Koreans.

In this study, patients who underwent orthodontic or orthognathic surgery were excluded because the movement of the anterior teeth would induce various morphometric changes (segmentation, unification, and changes in orientation, shape, and length) in the adjacent palatal rugae [8, 10, 16, 17]. Additionally, patients with maxillofacial deformities, congenital defects, or moderate crowding of more than 4 mm were excluded from the study because the growth and development of the palate, and subsequent formation of the dental arch, may also affect the direction of the rugae [20, 25].

The present study investigated the morphological features of the palatal rugae in South Korean individuals and found that 43.5% of the patients had three primary palatal rugae, and 36.1% had four. Approximately 11.1% of the patients had two or fewer primary palatal rugae, and the number of primary palatal rugae was not different between the right and left sides or between men and women. The difference in the mean number of secondary ruga between men and women was about 0.2, but it was not clinically relevant when deciding for superimposition. These findings were similar to those of a previous study [20, 21]. If the number of primary palatal rugae is less than two, the selection of a stable reference point in digital superimpositions is challenging.

This study also investigated the differences in the positions of the 3rd primary palatal rugae relative to the number of rugae. The results were very interesting; we identified significant differences in the positions of the 3rd palatal rugae according to the presence of additional posterior rugae. The proportion of patients with another primary or secondary ruga posterior to the 3rd primary palatal ruga was relatively high (78% for the right side and 72% for the left). In such cases, the proportions of cases in which the anterior limit of the 3rd palatal ruga was positioned anterior to the contact point between the first premolar and the second premolar were 88% and 80% for the right and left sides, respectively, whereas the proportions of cases in which the posterior limit was positioned anterior to the contact point between the first premolar and the second premolar were 25% and 24% for the right and left sides, respectively.

Conversely, in cases in which the 3rd primary palatal ruga was the most posterior ruga, the proportions were considerably lower. The proportions of cases in which the anterior limit was positioned anterior to the contact point between the first premolar and the second premolar were 40% and 57% for the right and left sides, respectively, and those in which the posterior limit was positioned posterior to the contact point between the first premolar and the second premolar were 2% and 1% for the right and left sides, respectively. In other words, the 3rd primary palatal ruga is located more significantly anteriorly when there is an additional ruga posterior to the 3rd primary palatal ruga.

The position of the rugae is an important element in their stability, with the more anteriorly positioned rugae more affected by anterior tooth movement [8, 10, 16, 17]. Thus, if the 3rd palatal ruga is located anteriorly, it is more likely to undergo a change in its position due to tooth movement during orthodontic treatment, and it is therefore unwise to use it as a reference area. In previous studies, the 3rd palatal ruga has been used as the reference area for digital superimpositions, regardless of its position. However, the results of the present study showed that the anteroposterior positions of the 3rd palatal ruga change depending on the presence of additional ruga posterior to the 3rd palatal ruga. Therefore, when superimposing digital models, this discrepancy needs to be considered when determining the suitability of using the 3rd palatal ruga as a reference area in premolar extraction treatment. If the 3rd palatal ruga is located anteriorly, it may be affected by tooth movement during orthodontic treatment with large retraction of the maxillary anterior teeth and may not be suitable as a reference area for superimposition before and after treatment. In this case, the cephalometric superimposition method may be more reliable than the digital model superimposition method when superimposing the results before and after treatment [17].

Several limitations to this study should be considered when interpreting the data. Because the sample of this study was mainly limited to Koreans in their lower 20s, it is difficult to extrapolate these findings to the general population. In addition, it is difficult to confirm whether the 3rd primary rugae that were located anteriorly in this study responded to orthodontic tooth movement due to the additional posterior rugae. Therefore, it is necessary to verify the accuracy of the digital model superimposition method by comparing it with the superimposition method using cephalometric radiographs or cone beam computed tomography.

## 5. Conclusions

In the present study, approximately 43.5% of the patients had three primary palatal rugae and 36.1% had four. When an additional primary or secondary ruga was posterior to the 3rd primary palatal ruga, the anterior and posterior limits of the 3rd primary palatal ruga were located more anteriorly compared to those in individuals without an additional posterior ruga. These observations suggest that the number of palatal rugae vary greatly in individuals, and that this affects the anteroposterior position of the 3rd primary palatal ruga. Therefore, when superimposing digital models, this discrepancy needs to be considered when determining the suitability of using the 3rd primary ruga as a reference area. When the 3rd primary ruga is located anteriorly, care should be taken when using it as a reference area for superimposition with a digital model before and after orthodontic treatment.

## Figures and Tables

**Table 1:** Mean number of the primary, secondary, and fragmentary rugae between right and left sides.

Type of ruga	Right (N = 343)		Left (N = 343)		*P *value
	Mean	SD	Mean	SD	
Primary ruga	3.5	0.8	3.4	0.6	0.064
Secondary ruga	1.3	0.8	1.2	0.8	0.103
Fragmentary ruga	1.0	0.9	1.3	1.2	< 0.001

SD: standard deviation.

**Table 2:** Mean number of the primary, secondary, and fragmentary rugae between sexes.

Type of ruga	Men (N = 110)		Women (N = 233)		*P *value
	Mean	SD	Mean	SD	
Primary ruga	3.4	0.7	3.4	0.6	0.838
Secondary ruga	1.1	0.6	1.3	0.6	0.024
Fragmentary ruga	1.0	0.8	1.1	0.7	0.229

SD: standard deviation.

**Table 3:** The frequency of the right and left primary rugae.

Number of primary rugae	Right (N = 343)		Left (N = 343)		Total	*P* value
	Number	%	Number	%	Mean %	
1	3	0.9	2	0.6	0.8	
2	30	8.7	41	11.8	10.3	
3	150	43.7	148	43.2	43.5	
4	120	35.0	129	37.6	36.1	0.083
5	35	10.2	23	6.8	8.5	
6	5	1.5	0	0	0.8	
Total	343	100	343	100	100	

**Table 4:** The position of the anterior or posterior limit of 3rd primary ruga according to the presence of additional rugae posterior to the 3rd primary ruga, based on the contact points between the first premolar and the second premolar.

The position of the 3rd primary ruga	Number of ruga = 3			Number of ruga > 3			
	Right	Left	Total	Right	Left	Total	*P* value
Anterior limit of the 3rd ruga							
Anterior	39 (57%)	35 (40%)	74 (48%)	212 (88%)	171 (80%)	383 (84%)	< 0.001
Posterior	29 (43%)	51 (60%)	80 (52%)	30 (12%)	43 (20%)	73 (16%)	

Posterior limit of the 3rd ruga							
Anterior	1 (1%)	2 (2%)	3 (2%)	60 (25%)	52 (24%)	112 (25%)	< 0.001
Posterior	67 (99%)	84 (98%)	151 (98%)	182 (75%)	162 (76%)	344 (75%)	

Total	68	86	154	242	214	456	

Anterior: anterior to the contact points between the first premolar and the second premolar; Posterior: posterior to the contact points between the first premolar and the second premolar.

## Data Availability

The data used to support the findings of this study are available from the corresponding author upon request.
